# Integrated traditional Chinese medicine and Western medicine strategies for the treatment of bronchiectasis: a comprehensive review

**DOI:** 10.1186/s13020-026-01324-0

**Published:** 2026-01-14

**Authors:** Yue Ou-Yang, Li-Xuan Zeng, Yang-Yang Xing, Hua Zhou, Qi-Biao Wu

**Affiliations:** 1 Faculty of Chinese Medicine and State Key Laboratory of Mechanism and Quality of Chinese Medicine, Macau University of Science and Technology, Macau, 999078 China; 2 Zhuhai M.U.S.T. Science and Technology Research Institute, Guangdong-Macao In-Depth Cooperation Zone in Hengqin, Zhuhai, 519000 Guangdong China; 3 Chinese Medicine Guangdong Laboratory (Hengqin Laboratory), Guangdong-Macao In-Depth Cooperation Zone in Hengqin, Zhuhai, 519000 China; 4 The Macau Holy House of Mercy, Macau, 999078 China

**Keywords:** Bronchiectasis, Traditional Chinese medicine, Western medicine, Integrated treatment, Personalized treatment

## Abstract

Bronchiectasis is a complex and heterogeneous disease with various etiologies and clinical manifestations. While Western medicine (WM) primarily focuses on infection control, symptom management, and airway clearance techniques, traditional Chinese medicine (TCM) adopts a holistic strategy aimed at systemic regulation and immune modulation through herbal formulae and acupuncture. The integration of TCM and WM offers a comprehensive therapeutic framework that targets both clinical manifestations and the underlying pathophysiology. This review systematically outlines current WM treatment strategies, such as antibiotic therapy, anti-inflammatory drugs, and surgical interventions. The TCM treatment principles, including individualized syndrome differentiation and treatment, specific TCM formulae, and acupuncture therapies, are detailed. This study further synthesizes clinical evidence demonstrating that integrated TCM-WM therapy not only significantly alleviates symptoms and improves lung function but also enhances immune regulation and quality of life. This combined strategy not only improves clinical outcomes but also enhances patients’ quality of life, which provides a more personalized and multidimensional paradigm to manage bronchiectasis. Future research should focus on optimizing integrated protocols, rigorous randomized controlled trials, and exploring novel therapeutic targets to consolidate the evidence base for this synergistic model.

## Introduction

**Fig. 1:**
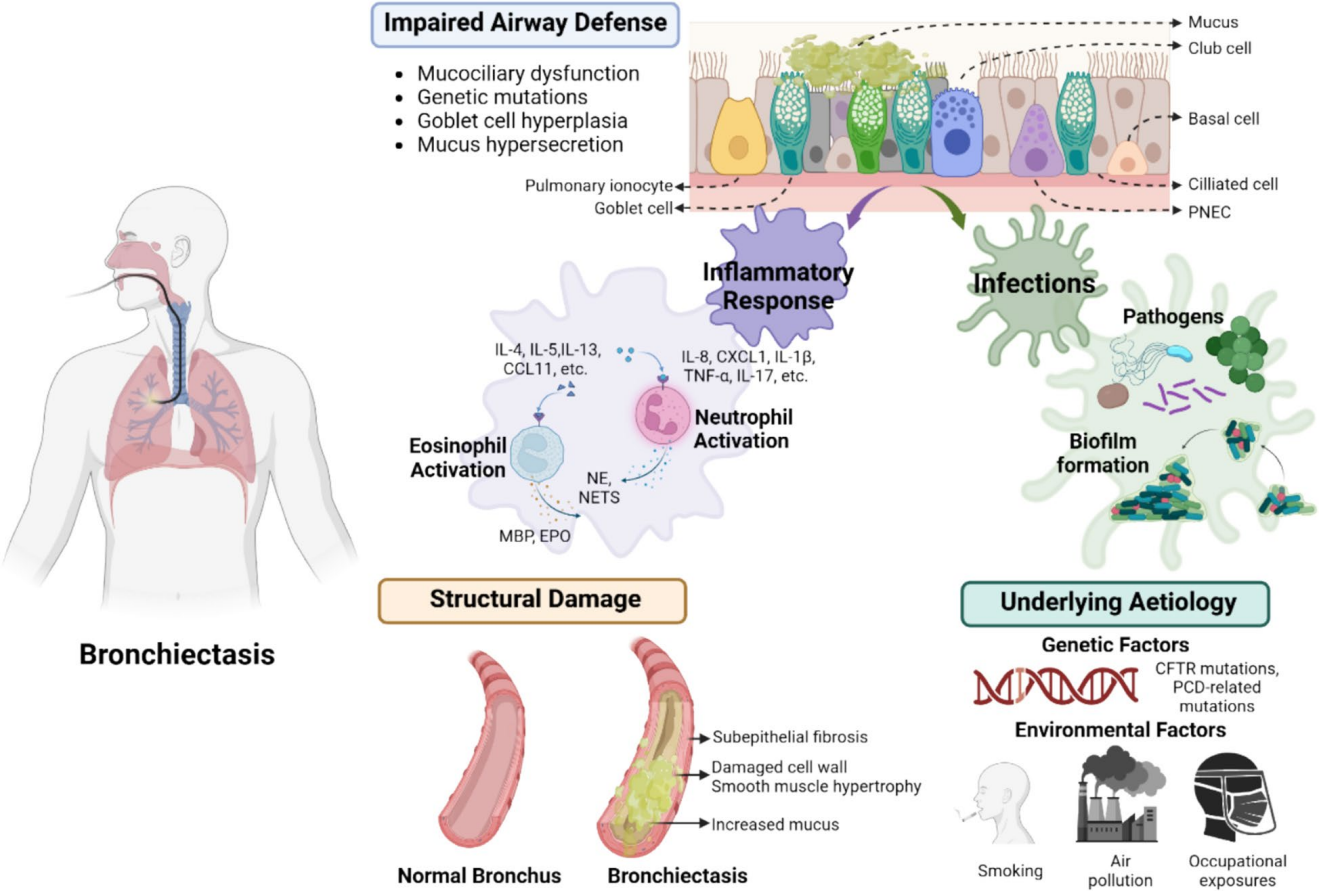
Pathophysiology of bronchiectasis

Bronchiectasis is a chronic respiratory disease characterized by irreversible bronchial dilation, imposing a substantial global health burden due to its heterogeneous etiology and persistent therapeutic challenges [1]. The pathological changes in bronchiectasis typically result from a combination of factors, such as previous respiratory infections, immune deficiencies, genetic predispositions, airway obstruction, and repeated aspiration, which initiate and sustain a vicious cycle of inflammation and airway remodeling. Patients often present with chronic cough, excessive sputum production, and hemoptysis, which severely impair quality of life [2]. Given the multifactorial etiology and heterogeneous nature of bronchiectasis (Fig. 1), treatment methods based solely on Western medicine (WM) or traditional Chinese medicine (TCM) have inherent limitations. With continuous advances in medical research, treatment strategies have evolved from simple symptom management to personalized approaches that target distinct clinical patterns and underlying mechanisms [3]. This review aims to comprehensively summarize the current treatments for bronchiectasis using both TCM and WM and to propose integrative, personalized treatment strategies that combine conventional clinical practices with modern mechanistic insights.

Chemokine (C-X-C motif) ligand 1 (CXCL1), tumor necrosis factor alpha (TNF-α), interleukin 1 beta (IL-1β), interleukin 4 (IL-4), interleukin 5 (IL-5), interleukin 17 (IL-17), interleukin 8 (IL-8), interleukin 13 (IL-13), C–C motif chemokine ligand 11 (CCL1), major basic protein (MBP), erythropoietin (EPO), neutrophil elastase (NE), pulmonary neuroendocrine cell (PNEC), transmembrane conductance regulator (CFTR), programmed cell death (PCD), and neutrophil extracellular traps (NETs).

This figure illustrates the "malignant vortex model" of bronchiectasis, highlighting the interconnected pathophysiological mechanisms that drive the progression of the disease. The central theme is the self-perpetuating cycle of airway dysfunction, inflammation, infection, and structural damage [4]. It also integrates the roles of genetic predispositions and environmental factors, emphasizing that bronchiectasis is a heterogeneous disease resulting from multiple converging etiological factors.

## Western medicine treatment for bronchiectasis

**Fig. 2:**
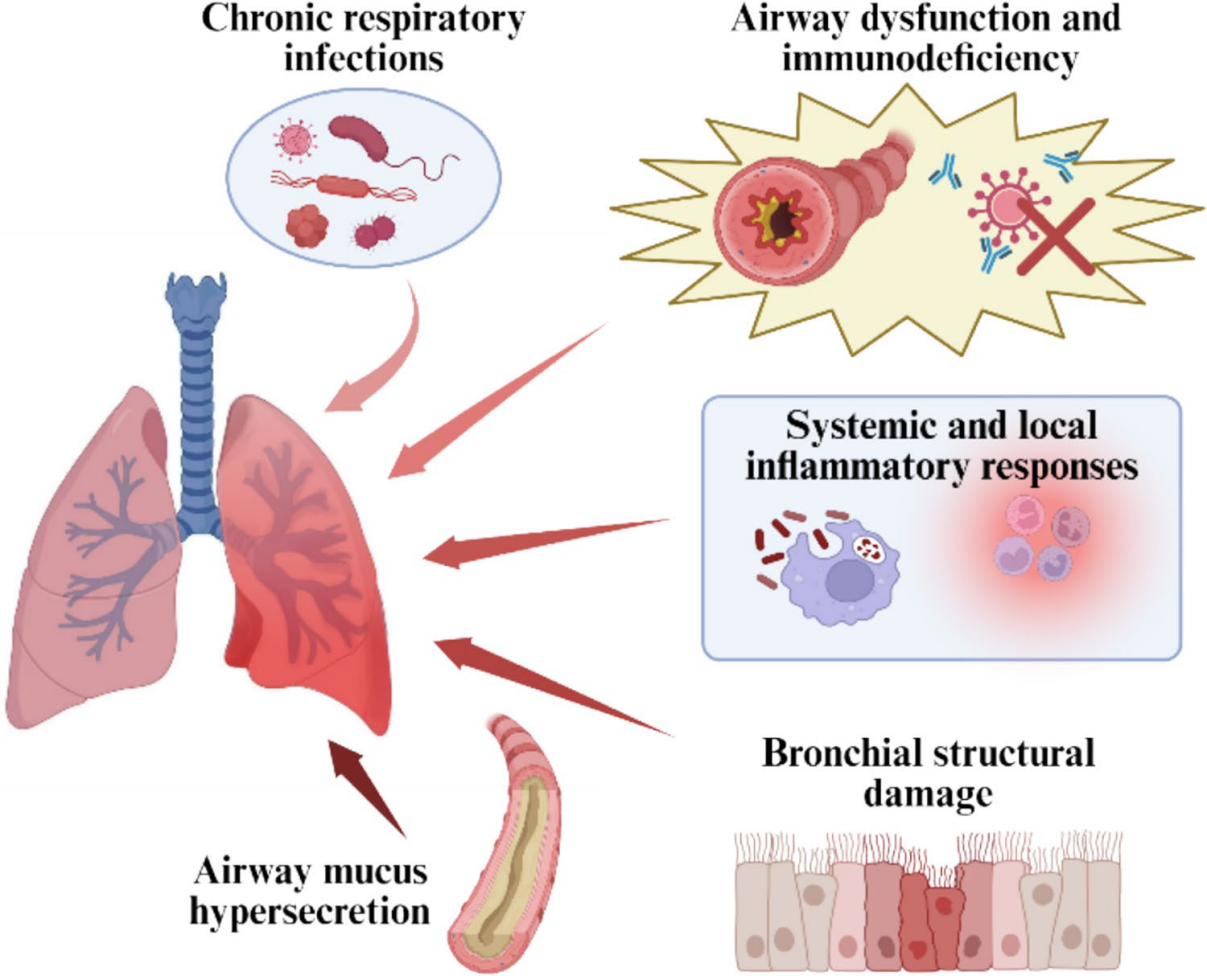
Core pathophysiological mechanisms of bronchiectasis

Bronchiectasis encompasses diverse pathophysiological mechanisms and clinical syndromes. WM treatment strategies for bronchiectasis aim to control infections, alleviate inflammation, and improve lung function. They include physical therapies to aid sputum clearance, drug therapies with antibiotics and antifungals, and immunomodulatory agents for patients with immunodeficiency. Eradicating or suppressing pathogenic microorganisms is crucial for preventing acute exacerbations. In some cases, surgical intervention may be necessary to remove damaged bronchial segments. Comprehensive care also requires the integrated management of acute exacerbations and disease-related complications (Fig. 2).

### Pathophysiology of bronchiectasis

#### Chronic respiratory infections

Chronic respiratory infections play a central and multifaceted role in bronchiectasis. The persistence of an infectious agent in the airways of patients with bronchiectasis results from multiple defects in the immune response and different microorganism defense mechanisms, leading to chronic bronchial infection [5]. These infections, often due to persistent pathogens such as *Pseudomonas aeruginosa*, *Haemophilus influenzae*, and *Streptococcus pneumoniae*, disrupt the normal airway environment [6]. Infections, particularly those caused by *P. aeruginosa*, induce airway inflammation, damage the epithelial barrier, and contribute to airway wall thickening and eventual dilation. Woo et al. conducted a 16-year follow-up study of 29 patients with bronchiectasis, confirming the significant stability of the microbiome. Chronic infections caused by *P. aeruginosa* or *H. influenzae* often last for many years, regardless of which treatment is used [7]. In clinical practice, chronic bacterial respiratory infection is often defined by the repeated isolation of the same microorganism from respiratory cultures over time [8, 9].

#### Airway dysfunction and immunodeficiency

**Fig. 3:**
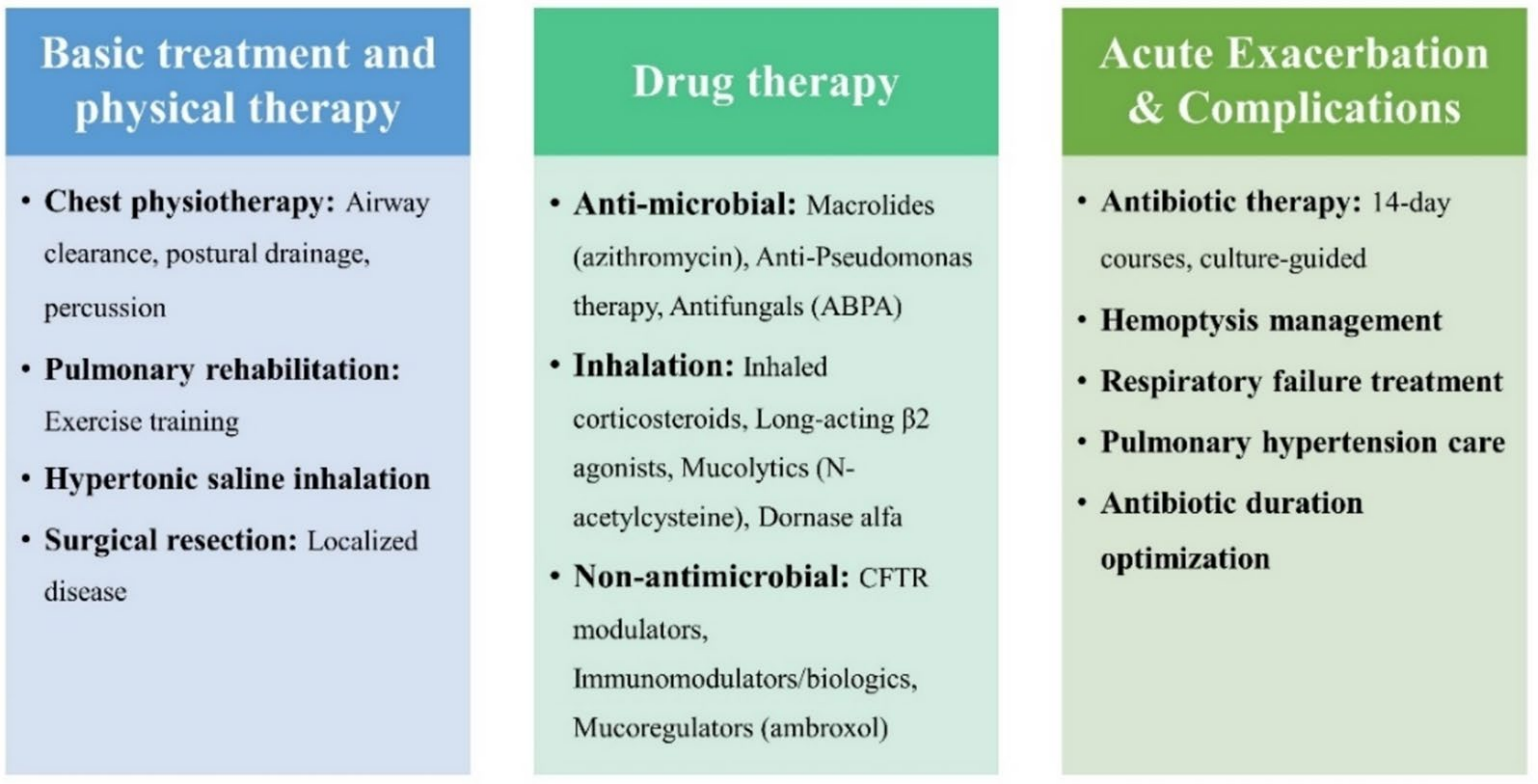
Current management strategies for bronchiectasis. This figure details current treatment strategies, covering basic treatment and physical therapy, drug therapy (antimicrobial, inhalation, and other nonantimicrobial approaches), and the management of acute exacerbations and complications

Structural or functional abnormalities of the airway constitute key etiological factors. Primary ciliary dyskinesia is a genetic disease that leads to mucus clearance disorders due to abnormal ciliary structure or function, which in turn leads to bronchiectasis [10]. This dysfunction results in the accumulation of mucus in the airways, creating an environment conducive to pathogen growth and causing recurrent infections that ultimately lead to bronchiectasis. Ciliary dysfunction leads to mucus accumulation, causing repeated infection and inflammation, eventually leading to bronchiectasis [11]. Moreover, cystic fibrosis (CF) is a genetic disease caused by conductance regulator (CFTR) gene mutation. The abnormal function of the CFTR protein affects mucus secretion and ion transport [12]. Abnormal CFTR function leads to mucus that is too viscous and difficult to remove, causing airway obstruction and chronic infection. In addition, some primary or secondary immunodeficiencies, such as common variable immunodeficiency (CVID) [13], hypogammaglobulinaemia, and chronic granulomatous disease, may lead to bronchiectasis [14, 15]. These immune deficiencies weaken the body’s defense against respiratory pathogens, increasing the susceptibility of airways to chronic infections. These immunocompromised patients are more susceptible to infections that are difficult to clear, leading to persistent airway inflammation and tissue damage [16], ultimately leading to bronchiectasis (Fig. 3).

#### Systemic and local inflammatory responses

Systemic and local inflammatory responses play significant roles in bronchiectasis. In primary ciliary dyskinesia, cystic fibrosis, or other immune deficiencies, chronic inflammation of the airways is a common outcome. This persistent inflammatory response damages airway structure, leading to tissue remodeling and exacerbating the progression of bronchiectasis. Systemic inflammation, such as rheumatoid arthritis, systemic lupus erythematosus, and other systemic diseases, affects the distal organs of the body and the airway, causing local airway inflammation and tissue damage. These changes often lead to bronchiectasis [17].

When the immune system is activated and many immune cells, such as neutrophils and macrophages, are recruited to the airways, the inflammatory mediators (e.g., IL-8, TNF-α) and proteases released by these cells further damage the airway wall, resulting in airway structural destruction and functional impairment. This inflammatory response is associated with airway dysfunction and immune deficiency, forming a vicious cycle that drives the progression of bronchiectasis. In addition, a notable eosinophilic inflammatory response is present in a subset of patients with bronchiectasis. The activation and accumulation of eosinophils may lead to airway inflammation and tissue remodeling, which represents an important pathological mechanism in bronchiectasis [18].

#### Bronchial structural damage

Bronchiectasis is a chronic respiratory disease often initiated by persistent airway irritation or injury [19]. For instance, severe gastroesophageal reflux disease (GERD) can induce or exacerbate bronchiectasis. Gastric acid reflux to the esophagus and larynx may cause airway inflammation and bronchial stimulation, which usually causes or aggravates bronchiectasis [20]. Moreover, dysphagia can cause food or saliva to stray into the airway, triggering chronic airway inflammation and infection and increasing the risk of bronchial injury [21, 22]. Vocal cord dysfunction may also hinder the normal clearance of airway secretions and increase the possibility of respiratory tract infection, thereby increasing the risk of bronchiectasis.

Smoke inhalation can introduce a variety of toxins that can damage the airway epithelium and disrupt mucociliary clearance, whether from tobacco or from environmental sources. This cycle results in progressive damage to the bronchial structure, impairing lung function and worsening the patient’s condition. This makes the bronchi more susceptible to infections, airway inflammation, and damage caused by acute smoke inhalation, which occurs repeatedly and may also cause chronic damage to and remodeling of the bronchial wall [23, 24]. In addition, whether caused by intracavitary foreign bodies or enlarged lymph nodes outside the cavity, airway obstruction may lead to insufficient ventilation and chronic inflammation, further damaging the bronchial wall.

#### Airway mucus hypersecretion

Airway mucus hypersecretion is a key pathophysiological feature of bronchiectasis that significantly affects disease progression and patient prognosis. Under normal conditions, mucus in airways effectively traps inhaled particles and pathogens, which are then cleared by a mucociliary escalator. In bronchiectasis, however, this balance is disrupted by chronic inflammation, often triggered by persistent infections from pathogens [25]. This inflammatory state induces goblet cell hyperplasia and submucosal gland hypertrophy within the airway epithelium, resulting in the overproduction of mucus that is abnormally thick and viscous [26]. This excessive and abnormal mucus obstructs the airways, impairs airflow, and reduces lung function. It also provides a favorable environment for pathogen colonization and growth, exacerbating the cycle of infection and inflammation [27]. Furthermore, the increased mucus burden can hinder the effective delivery of antibiotics to the site of infection [28]. Understanding the mechanisms underlying airway mucus hypersecretion is crucial for developing targeted therapies to reduce mucus production, improve mucus clearance, and ultimately enhance the management of bronchiectasis [29].

### Current treatment strategies for bronchiectasis

#### Basic treatment and physical therapy

The management of bronchiectasis fundamentally focuses on enhancing respiratory tract cleansing and facilitating sputum expectoration, thereby mitigating infection and inflammation. A hallmark of bronchiectasis is excessive airway mucus production coupled with impaired clearance, resulting in mucus stasis. The inhalation of hypertonic solutions, such as hypertonic saline, can augment the efficacy of physical therapy. Notably, however, current guidelines advise against the routine use of inhaled recombinant human DNase in adult patients with bronchiectasis [30].

Physical modalities, including chest physiotherapy [31] and airway cleaning techniques [32] (postural drainage, chest percussion, etc.), are foundational components of care. These approaches mitigate the risk of airway obstruction and subsequent infections by assisting patients in more effectively expectorating sputum. Moreover, contemporary guidelines and clinical appraisals of bronchiectasis frequently advocate the integration of pulmonary rehabilitation (PR) and/or exercise training (ET) into therapeutic regimens [33–35]. PR is designed to enhance patients’ overall health and exercise capacity through a multifaceted approach that incorporates exercise regimens and respiratory education [36]. For a select subset of patients with localized, severe disease refractory to medical therapy, surgical resection of the affected bronchial segments may be considered as a potential option to reduce the focus of chronic infection and improve lung function [37].

#### Drug therapy

##### Antimicrobial therapy

Antimicrobial therapy is essential for managing infections in bronchiectasis, particularly during acute exacerbations. The long-term use of macrolide antibiotics such as azithromycin has been shown to reduce the frequency of exacerbations by targeting pathogens and modulating inflammation. For example, a meta-analysis of randomized controlled trials (RCTs) revealed that azithromycin treatment significantly improved the rate of exacerbation-free periods and reduced the number of pulmonary exacerbations in noncystic fibrosis bronchiectasis patients [38].

Antifungal therapy is also important, especially for conditions such as allergic bronchopulmonary aspergillosis (ABPA), which involves the use of antifungal drugs and steroids to control allergic reactions and inflammation [39–41].

For specific pathogens, targeted eradication therapy is a key strategy [42, 43]. For example, ciprofloxacin 500 mg (twice daily) is recommended for oral treatment for two weeks in patients with bronchiectasis who were first isolated from bacteria and progressed [44]. For refractory or chronic infections, a more intensive regimen may be considered, such as a 2-week course of intravenous antipseudomonal agents (e.g., an aminoglycoside combined with a β-lactam antibiotic), potentially followed by long-term suppressive therapy with inhaled antibiotics such as tobramycin or polymyxin [45–47]. This staged approach aims to reduce the bacterial load, alleviate inflammation, and prevent recurrence.

##### Inhalation therapy

Inhalation therapy is a key component of bronchiectasis management, particularly for patients with reversible airway obstruction. Inhaled corticosteroids (ICSs) and long-acting β_2_ agonists are commonly used to reduce airway inflammation and improve lung function [48]. However, these treatments should be used with caution in patients without asthma or chronic obstructive pulmonary disease (COPD) [23]. Inhaled mucolytics, such as N-acetylcysteine (NAC), reduce mucus viscosity and enhance clearance, whereas dornase alfa degrades DNA in purulent mucus, improving mucus clearance and reducing retention [49].

##### Other nonantimicrobial therapies

In addition to antimicrobial and inhalation therapy, other nonantimicrobial therapies play a significant role in the management of bronchiectasis. CFTR modulators are specifically used to treat CF-related bronchiectasis by improving CFTR protein function [50, 51]. Immunomodulatory therapy, including immunoglobulin replacement therapy and biological agents such as anti-TNF-α drugs, is effective in patients with bronchiectasis associated with immunodeficiency and autoimmune diseases [52]. Mucoregulators, including mucolytics such as ambroxol and acetylcysteine, and mucokinetics such as tromethamine, are intended to facilitate mucus clearance and help alleviate airway obstruction [53]. Additionally, anti-inflammatory agents such as nonsteroidal anti-inflammatory drugs (NSAIDs) (e.g., ibuprofen and naproxen) [54] and COX-2 inhibitors (e.g., celecoxib) [55] are used to reduce airway inflammation and alleviate discomfort.

#### Treatment of acute exacerbations and complications

The management of bronchiectasis focuses not only on long-term control but also on addressing acute exacerbations and associated complications. Acute exacerbations, which are frequently precipitated by respiratory infections, are characterized by heightened neutrophilic airway inflammation and can lead to accelerated lung function decline and worsened quality of life [56]. Therefore, when treating bronchiectasis, patients are often advised to prevent the disease from entering acute exacerbations.

The treatment of acute exacerbation of bronchiectasis requires comprehensive management strategies, of which antimicrobial therapy is the core. Before the availability of sputum culture and sensitivity results, empirical antibiotic therapy is initiated. The choice of agent should be guided by the patient’s prior microbiological history (e.g., known colonization with* P. aeruginosa*) or local epidemiological data on common pathogens [57]. In addition, for possible complications of bronchiectasis, such as hemoptysis [58], chronic respiratory failure [59], and pulmonary hypertension [60], corresponding treatment measures need to be taken, which may include drug therapy, interventional therapy, or oxygen therapy.

Although antibiotics are the mainstay treatment for bronchiectasis exacerbations, the course of antibiotics should not be too long. The results revealed that extending the duration of antibiotic treatment to intravenous courses beyond 14 days did not consistently reduce the clinical bacterial load [61], indicating that treatment efficacy is not solely dependent on prolonged administration.

## TCM treatment for bronchiectasis

Bronchiectasis is a chronic, intractable disease with a prolonged course and repeated exacerbations. The common symptoms of bronchiectasis are cough, purulent sputum, and intermittent hemoptysis, which are often accompanied by chest pain, chest tightness, wheezing, fatigue, and other discomfort. TCM has distinct characteristics and advantages in alleviating symptoms, shortening the course of the disease, and reducing the recurrence rate of bronchiectasis. Under the guidance of the basic theory of TCM, the fundamental pathogenesis of bronchiectasis is “deficiency in origin and excess in superficiality,” with deficiency of healthy qi (e.g., lung qi, spleen qi, kidney qi, or yin deficiency) underlying susceptibility, whereas phlegm, heat, and blood stasis act as pathogenic products often precipitated by exogenous factors [62].

### TCM syndromes and treatment principles

According to TCM theory, bronchiectasis can be classified into various syndrome types to address patients’ diverse pathological states, as outlined in the *Guidelines for Integrated Chinese and Western Medicine Treatment of Bronchiectasis* [63]. Modern research further categorizes these patterns into syndrome patterns, such as *liver fire invading the lung*, *phlegm turbidity obstructing the lungs*, *phlegm and blood stasis obstructing the lungs*, *dryness-heat injuring the lungs*, *yin deficiency with excess fire*, and *qi deficiency with blood overflow* [64]. Syndrome differentiation and individualized treatment form TCM’s core principles in disease understanding and clinical management. These syndromes are closely related to the etiology and pathogenesis of bronchiectasis. In TCM clinical practice, the treatment process is generally divided into acute and remission stages, which are the two main phases [65]. In the pathogenesis of bronchiectasis, phlegm heat, phlegm dampness, qi deficiency, and other pathological factors often do not exist alone but coexist and interrelate, mixed with deficiency and excess. On the basis of TCM theories, clinical treatment principles are developed for different symptoms, and corresponding TCM formulae are prescribed for patients. Table 1 summarizes the key syndrome types of bronchiectasis from a TCM perspective, outlining the syndrome type, disease stage, core symptoms, and representative TCM formulae.

**Table 1:** Classification and management of bronchiectasis based on TCM syndrome differentiation

Syndrome type	Disease stage	Main clinical symptoms	Representative TCM formula
Lung phlegm-heat congestion	Acute exacerbation/Stable stage	Phlegm (yellow, thick, purulent), fever and thirst, constipation, red tongue with yellow or greasy coating, rapid and slippery pulse	*Qingjin Huatan* decoction [66], *Qianjin Weijing* decoction [67], *Zhikuoning* mixture [68], *Bupi Qingfei* decoction [69], *Sangbaipi* decoction [70], *Kuandonghua* formula [71], *Qingjin Beilou* decoction [72]
Lung phlegm-dampness stagnation	Acute exacerbation	Phlegm (white, thick, purulent), fullness, poor appetite, heaviness of the whole body, greasy white fur, slippery pulse	*Cangma* pills [73], *Erchen* tang [74], Gleditsiae pills [75]
Liver fire invading the lung	Acute exacerbation/Stable stage	Cough, light white sputum, fullness in the abdomen, irritability, thin white tongue coating, slippery or stringy pulse	*Xiebai* san [76], *Daige* san [77]
Collateral damage and hemoptysis	Acute exacerbation	Hemoptysis, blood in phlegm	*Yunnan Baiyao* capsules [78], *Xijiao Dihuang* decoction [79], *Luohuazizhu* granules [80]
Lung qi and yin deficiency	Stable stage	Dry cough, little phlegm, sticky phlegm that is difficult to cough up, fatigue, spontaneous sweating, night sweats, hot hands and feet, thirst, pale or red tongue, deep or weak pulse or fine pulse	*Buzhong Yiqi* decoction [81], *Sangbaipi* decoction [82], *Wanfei* yin [83]
Lung and spleen deficiency	Stable stage	Phlegm (white and thin), shortness of breath, spontaneous sweating, poor appetite, fatigue, fullness, abdominal distension, loose stools, enlarged tongue or tooth-marked tongue, deep and thin pulse or slow pulse or weak pulse	*Buzhong Yiqi* decoction [81], *Liujunzi* decoction [84]
Fire excess from yin deficiency	Acute exacerbation/Stable stage	Cough, little phlegm, low fever, night sweats, dry mouth, red tongue with little coating, weak pulse	*Baihe Gujin* decoction [85]

### TCM formulae and clinical evidence

TCM not only aims to relieve clinical symptoms and reduce the frequency of acute exacerbations but also contributes to regulating immune function and reducing inflammation, thereby improving lung function and patients’ quality of life [86]. These TCM formulae can not only relieve symptoms but also adjust and restore the overall balance to achieve disease control in patients with bronchiectasis. The most widely used TCM prescriptions include *Qingjin Huatan* decoction [87], *Qianjin Weijing* decoction [88], *Buzhong Yiqi* decoction, *Liujunzi* decoction, etc. In addition, there are many Chinese patent medicines (CPMs), such as the *Tanreqing* injection and the *Zhikuoning* mixture [89]. The *Zhikuoning* mixture, developed by the esteemed TCM practitioner Professor Cao Shihong for the pattern of lung phlegm-heat congestion, serves as an example. Reported data from clinical studies indicate a high rate of clinical response with this CPM. Research suggests that it may be associated with reductions in neutrophil and lymphocyte counts, as well as in the levels of inflammatory and remodeling mediators including IL-8, TNF-α, MMP-9, NE, and collagen III/IV, suggesting to a potential role in ameliorating airway inflammation and remodeling [68, 90].

Other clinical investigations provide supporting evidence. A study by Zhang et al*.* [91] reported that an herbal formula was associated with a decrease in the severity and frequency of cough and sputum. Research on the *Qianjin Weijing* decoction revealed a correlation between its use and improvements in lung function parameters such as forced expiratory volume in one second (FEV₁) and forced vital capacity (FVC) [92]. TCM therapies have also been shown to enhance immune function. Furthermore, Ma et al. [93] reported that TCM therapies are also associated with increased levels of immune markers, such as CD4^+^ and CD8^+^ T cells, in patients receiving TCM treatment.

With the advent of big data analytics, data mining has supported pattern recognition in TCM prescriptions for bronchiectasis [94]. However, it is important to note the current limitations of the evidence base. A significant portion of the clinical support for TCM in this field originates from observational studies, case reports, and expert opinions, with a relative paucity of large-scale, high-quality RCTs [95]. The inherent empiricism and individualization of TCM, coupled with variability in practice, present substantial challenges for standardizing interventions and reproducing research outcomes. These factors highlight a clear need for future work to develop more consistent syndrome differentiation frameworks and to establish treatment protocols through rigorously designed clinical trials.

### Acupuncture therapy

TCM has shown notable clinical efficacy in treating bronchiectasis. Among its various modalities, acupuncture significantly alleviates symptoms such as cough, sputum production, chest tightness, and hemoptysis. As a key therapeutic approach in TCM, its clinical application may help alleviate characteristic symptoms such as cough, sputum production, chest tightness, and hemoptysis [96]. From the perspective of TCM theory, bronchiectasis is often associated with phlegm and dampness obstructing the lung meridians. Acupuncture helps to unblock these meridians, clear phlegm and dampness, and prevent further pathological accumulation. Through the regulation of qi and blood circulation, acupuncture improves systemic function and mitigates stagnation, thus relieving key symptoms such as productive cough and chest discomfort [97]. In current clinical practice, acupuncture is frequently integrated with herbal prescriptions to treat patients. This combined approach is employed during both acute exacerbations and stable phases, with reports indicating that it may support improvements in pulmonary function, quality of life, and overall recovery [98, 99]. In addition, the different acupoints chosen for acupuncture treatment can reflect individualized syndrome differentiation and treatment. Clinical treatment can formulate targeted and effective treatment strategies based on the specific pathogenesis of the patient.

## Integrated TCM-WM treatment for bronchiectasis

Bronchiectasis represents a persistent challenge in clinical management. Evidence from practice indicates that when WM or TCM is applied independently, clinical outcomes often remain suboptimal. Integrated TCM and WM strategies play important roles in the treatment of bronchiectasis and are gaining increasing attention and application in clinical practice. It establishes personalized strategies that holistically address the multifaceted pathophysiology of bronchiectasis—including recurrent infection, inflammatory dysregulation, and impaired mucociliary clearance. The primary objective of this integrated strategy is to improve the main contradictions currently faced in clinical practice, especially the treatment and serious decline in the quality of life of patients with acute exacerbation.

### Current challenges and clinical bottlenecks

Despite a growing research interest in bronchiectasis over the past decade, its clinical management remains challenging. The disease is highly heterogeneous, with substantial variation in etiology, clinical course, and prognosis among patients. Treatment guidelines also vary regionally, reflecting differences in clinical practice and resource availability [100]. Delays in pharmacological intervention, insufficient attention during early-stage disease, and untimely pulmonary rehabilitation often cause patients to miss the optimal treatment window. Consequently, a substantial proportion of patients progress to severe disease, which is associated with a high 4-year all-cause mortality rate [101].

The clinical management of bronchiectasis continues to present significant challenges, primarily owing the inherent limitations of any single therapeutic system in addressing the multifaceted pathophysiological complexity of the disease. WM offers rapid control of acute infections and airway obstruction [34, 102], and its interventional strategy focuses primarily on pathogen eradication and symptomatic management. This approach shows limited efficacy in modulating immune homeostasis, preventing disease recurrence, or achieving sustained long-term improvement in quality of life. In contrast, TCM adopts a holistic therapeutic paradigm. Through syndrome differentiation and treatment, it has the potential to alleviate symptoms, regulate systemic balance, and promote disease stabilization. However, TCM generally acts more slowly in acute infection, and its mechanisms of action have not been fully elucidated by modern scientific standards. The lack of standardized efficacy evaluation criteria also limits its broader application [103, 104]. The distinct and complementary profiles of these limitations within each system highlight the rationale and clinical need for developing integrated TCM-WM strategies. This approach aims to synergize the rapid interventional capacity of WM with the systemic regulatory and long-term stabilizing potential of TCM.

### Potential mechanisms of TCM-WM synergy

The complex and interconnected pathophysiology of bronchiectasis, characterized by persistent infection, chronic inflammation, and progressive structural damage, necessitates a combined therapeutic approach. The integration of TCM and WM offers a uniquely complementary strategy that addresses both acute manifestations and underlying systemic dysregulation. WM provides rapid, targeted control of active infections through antimicrobial agents and specific suppression of key inflammatory mediators, effectively reducing pathogen load and mitigating acute exacerbations. In contrast, TCM operates through multi-target mechanisms that promote immune recalibration, support tissue repair, and facilitate holistic functional recovery. This functional and temporal complementarity enables seamless integration of both systems, establishing a foundation for synergistic treatment of the multifaceted pathology of bronchiectasis.

Substantial synergistic benefits emerge in terms of anti-infection and immunomodulation effects through TCM-WM synergy. While western antibiotics directly and effectively eliminate pathogens, the ongoing challenge of multidrug resistance remains insufficiently addressed. However, adjunctive TCM therapy may facilitate a reduction in antibiotic usage. In this context, TCM employs heat-clearing and detoxifying drugs such as *Scutellaria baicalensis* [105], *Lonicera japonica* [106], *and Forsythia suspensa* [107], which not only mitigate lung injury by suppressing both systemic and local inflammation [108], but also contain specific active components capable of disrupting biofilm structures and inhibiting microbial pathogenicity, thereby accelerating the clearance of infectious agents. Luo et al. demonstrated that the combination of baicalin with antibiotics significantly reduced the number of colony-forming units in implants, with efficacy markedly superior to that of antibiotic monotherapy [109]. Concurrently, TCM involves blood-activating and stasis-resolving drugs such as *Salvia miltiorrhiza*, *Curcuma aromatica*, and *Ligusticum chuanxiong* to modulate the inflammatory network and prevent tissue damage resulting from excessive immune responses [110]. Wu et al. reported that zedoary turmeric oil injection (ZTOI) suppressed the mRNA expression of IL-10 and IL-6 and alleviated pathological changes in the lungs, thereby exerting anti-inflammatory effects [111].

In addition to acute phase management, TCM also incorporates tonifying drugs such as *Astragalus membranaceus* [112], *Atractylodes macrocephala* [113], and *Ophiopogon japonicus* [114], which contribute to restoring the respiratory immune barrier and reducing pathogen colonization, thereby fundamentally decreasing the frequency of recurrent infections. Research has indicated that pretreatment with astragalus polysaccharide (APS) alleviates pathological changes induced by lipopolysaccharide (LPS) exposure. While reducing CD86 expression, the combined application of APS and LPS further increased the number of macrophages expressing the high mannose receptor C-type 1 (MRC1), substantiating the role of APS in protecting the host from inflammatory lung injury [115].

Airway structural defense enhancement represents another dimension of TCM-WM synergy. While WM directly addresses mucus retention through bronchodilators, ICSs, expectorants, and physical clearance techniques [116], TCM improves intrinsic airway self-cleaning capacity by regulating mucus secretion and enhancing ciliary function. Du et al. reported that *Pinellia ternata* significantly protects against ICS withdrawal effects by suppressing extracellular signal-regulated kinase (ERK) activation, consequently inhibiting goblet cell hyperplasia and MUC5AC gene overexpression, thus preventing post-ICS mucus hypersecretion and airway inflammation [117]. Furthermore, although established bronchiectasis is largely irreversible, early intervention with TCM may delay the process of airway remodeling [103]. Zou et al*.* reported that *Xuanfei Pingchuan* Prescription upregulated the protein and mRNA expression levels of SnoN and E-cadherin, thereby inhibiting epithelial-mesenchymal transition (EMT) and potentially targeting airway remodeling [118].

**Fig. 4:**
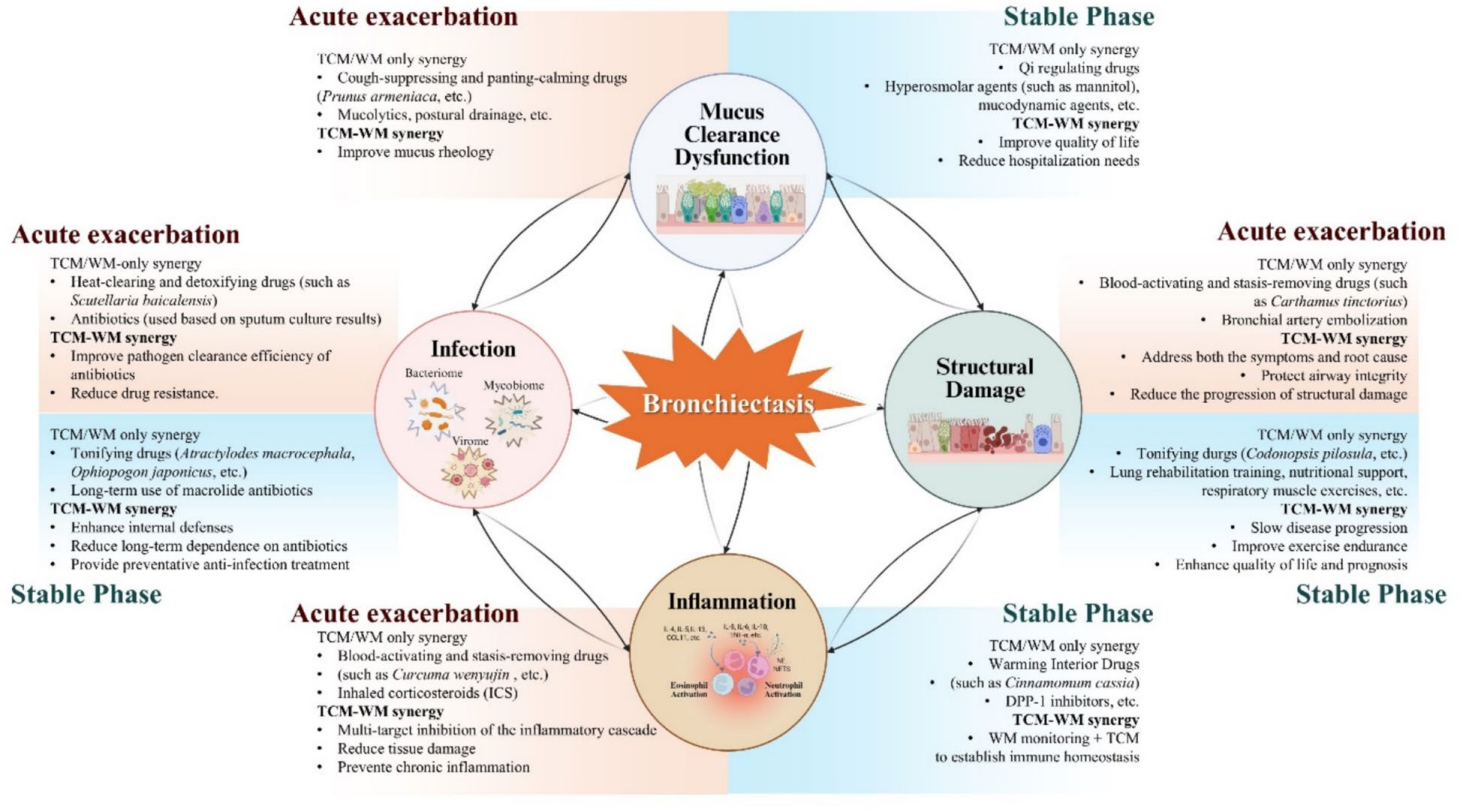
Therapeutic framework of integrated TCM-WM synergy for bronchiectasis treatment. This figure illustrates the integrated TCM-WM synergy strategy for disrupting the self-perpetuating “vicious vortex” of bronchiectasis, which is driven by four interconnected pathophysiological processes: infection, inflammation, mucus clearance dysfunction, and structural damage. The framework delineates the distinct yet complementary roles of TCM and WM treatments across both the acute exacerbation and stable phases of the disease. During acute exacerbations, WM enables rapid control of infection and inflammation, meanwhile TCM adjunctively enhances therapeutic efficacy and mitigates tissue injury. In the stable phase, TCM focuses on fortifying host defenses and promoting structural repair, whereas W treatment maintains disease control through long-term anti-inflammatory agents and airway clearance techniques. The model highlights key synergistic interactions between these treatment modalities, demonstrating how their combined multi-target strategy provides a comprehensive therapeutic approach to break the core disease cycle

In conclusion, the multifaceted mechanisms of TCM-WM synergy encompass anti-infection, immunomodulation, and airway defense, which collectively support the rationale for delivering comprehensive clinical management in bronchiectasis. These synergistic interactions, which form the theoretical foundation for integrated treatment strategies, are graphically summarized in Fig. 4, delineating the complementary roles of TCM and WM in disrupting the “vicious vortex” of bronchiectasis across both acute exacerbation and stable disease phases.

### Clinical evidence and integrated management strategies

The conventional management of bronchiectasis primarily involves WM and physical therapy to address the underlying pathology, control infection, and promote airway clearance [119]. In contrast, TCM employs a holistic approach centered on syndrome differentiation, which may offer benefits in alleviating acute symptoms and extending periods of stable remission. A growing body of clinical research over the past decade suggests that integrating TCM approaches into management strategies may improve overall symptom control and therapeutic outcomes for patients with bronchiectasis.

To systematically evaluate these benefits, Table 2 summarizes the clinical evidence supporting integrated TCM-WM treatment for bronchiectasis. For example, one observational study reported a higher clinical response rate with integrated TCM-WM treatment (95.0%) than with WM alone (80.0%), suggesting the potential for improved outcomes [149]. This synergy is particularly evident in clinical scenarios where conventional therapies reach their limitations.

**Table 2:** Clinical evidence on integrated TCM-WM treatment for bronchiectasis

Formula name	TCM source	Disease stage	Study detail (N = total participants)	Main findings of TCM-WN treatment	References
*Qingjin Huatan* decoction	14 TCMs	Acute exacerbation	- N = 130 - Control group (antibiotics plus drainage) versus experimental group (control therapy plus *Qingjin Huatan* decoction) - 7-day treatment	- Lower symptom scores ^*a^ - Reduced systemic inflammation markers (WBC, CRP, PCT) ^*a^ - Improvement in pulmonary function (FEV_1_, FVC, PEF) ^*a^ - Lower sputum cytokine levels (TNF-α, IL-6, IL-8) ^*a^ - Reduced protease activity (NE, Cathepsin G) ^*a^	[95]
		Acute phase	- N = 110 - Control group (conventional WM) versus experimental group (conventional WM treatment plus modified *Qingjin Huatan* decoction) - 1-month treatment	- Higher total effective rate (89.1% vs. 70.91%)^*b^ - Greater improvement in most symptoms (except fever)^*b^ - Greater reduction in WBC, NEUT, and CRP levels^*b^	[120]
		Bronchiectasis with co-existing infection	- N = 82 - Control group (conventional therapy, bronchoscopic alveolar lavage) versus experimental group (control therapy plus modified *Qingjin Huatan* decoction) - 2-week treatment	- Lower TCM symptom scores^*b^ - Higher total effective rate (95.12% vs. 78.05%)*b - Greater reduction in TNF-α and hs-CRP levels*b - Greater increase in IL-2 and greater reduction in TGF-β and IFN-γ levels^*b^ - Greater reduction in IL-23, IL-4, and IL-5 levels^*b^	[121]
			- N = 98 - Control group (basic therapy, alveolar lavage) versus experimental group (control therapy plus *Qingjin Huatan* decoction) - 2-week treatment	- Higher total effective rate (95.92% vs. 81.63%)^*b^ - Greater improvement in pulmonary function (PEF, FVC, FEV_1_)^*b^ - Greater reduction in inflammatory mediators (TNF-α, Hs-CRP, IL-6)^*b^ - Improved immune function (higher CD4^+^, CD4^+^/CD8^+^; lower CD8^+^)^*b^	[122]
*Qingfei Huatan* decoction	11 TCMs	Acute phase	- N = 80 - Control group (conventional WM) versus experimental group (conventional WM treatment plus *Qingfei Huatan* decoction) - 14-day treatment	- Superior clinical efficacy (97.5% vs. 80.0%)^*a^ - Significant improvement in pulmonary function (FEV_1_, FEV_1_/FVC) ^*a^ - Marked reduction in symptom scores and inflammatory markers (WBC, NEUT) ^*a^	[123]
			- N = 140 - Control group (antitussive, expectorant, antiasthmatic) versus experimental group (control therapy plus *Qingfei Huatan* decoction) - 14-day treatment	- Higher total effective rate (94.29% vs. 82.86%)^*a^ - Greater reduction in symptom scores (cough, sputum, hemoptysis, fever) ^*a^ - Improved lung function parameters (FEV_1_, FEV_1_/FVC) ^*a^	[124]
			- N = 60 - Control group (basic therapy) versus experimental group (basic therapy plus *Qingfei Huatan* decoction); - 10-day treatment	- Higher total effective rate^*b^ - Greater reduction in cough symptom scores and 24-h sputum volume^*b^ - More significant improvement in sputum composition (MUC5AC, NE) and serum inflammatory markers (CRP, IL-6, PCT)^*b^	[125]
*Weijing Erchen* decoction	13 TCMs	Acute phase	- N = 62 - Control group (conventional WM) versus experimental group (conventional WM treatment plus *Weijing Erchen* decoction) - 14-day treatment	- Lower symptom scores (cough, yellow sputum, hemoptysis, fever) ^*a^ - Reduced inflammatory parameters (NLR, WBC, CRP)^*b^ - Improved lung function (FEV_1_, FVC, FEV_1_/FVC)^*b^ - Higher efficacy rate (90.32% vs. 67.74%) ^*a^	[126]
*Weijing Xiaoyong* decoction	9 TCMs	Acute exacerbation	- N = 98 - Control group (non-pharmacological intervention, levofloxacin hydrochloride injection) versus experimental group (control therapy plus *Weijing Xiaoyong* decoction) - 14-day treatment	- Better pulmonary function and blood gas parameters ^*a^ - Lower clinical symptom scores ^*a^ - Superior treatment effectiveness (95.92% vs. 79.59%) ^*a^ - Favorable MMP-9/TIMP-1 profile modulation ^*a^	[127]
*Yulou Weijing* decoction	12 TCMs	Bronchiectasis with co-existing infection	- N = 80 - Control group (conventional WM) versus experimental group (conventional WM treatment plus *Yulou Weijing* decoction) - 3-week treatment	- Superior overall response rate (90% vs. 75%)^*b^ - More significant reduction in inflammatory markers (WBC, NEUT, CRP)^*b^ - Greater improvement in pulmonary function (FEV_1_, FEV_1_/FVC, PEF)^*b^	[128]
*Xiefei Huatan* decoction	10 TCMs	Acute exacerbation	- N = 120 - Control group (basic therapy, mucolytic agent, acetylcysteine solution) versus experimental group (control therapy plus *Xiefei Huatan* decoction) - 14-day treatment	- Higher total effective rate (91.67% vs. 78.33%)^*b^ - Greater reduction in TCM syndrome scores (cough, sputum, hemoptysis, etc.)*^*b^ - Better improvement in lung function parameters (FVC%pred, FEV_1_%pred, FEV_1_/FVC, DLCO%pred)^*b^ - More pronounced reduction in serum inflammatory markers (PCT, IL-6, CRP)^*b^ - Improved exercise capacity (FEV1%pred, 6MWT) and better quality of life scores (mMRC, BODE)^*b^	[129]
	13 TCMs		- N = 80 - Control group (conventional therapy) versus experimental group (WM-alone treatment plus *Xiefei Huatan* formula) - 14-day treatment	- Higher total effective rate (92.5% vs. 70%)^*b^ - Greater improvement in blood gas parameters (PaO₂, SaO₂, PaCO₂) ^*b^ - More significant reduction in serum inflammatory markers (CRP, IL-6, TNF-α)^*b^ - Enhanced immune function (increased CD3⁺, CD4⁺, CD4⁺/CD8⁺ ratio; decreased CD8⁺)^*b^	[130]
Modified *Jiawei Jiegeng* decoction	13 TCMs	Stable phase	- N = 60 - Control group (conventional therapy) versus experimental group (conventional therapy plus modified *Jiawei Jiegeng* decoction) - 1-month treatment	- Greater reduction in clinical symptom scores (fever, expectoration, cough, and moist rales)^*b^ - More favorable improvement in BALF inflammatory parameters (leukocyte count, lymphocyte%, neutrophil%, IL-6)^*b^ - Enhanced pulmonary function measures (FVC, FEV_1_, FEV_1_/FVC)^*b^	[131]
*Juqin* decoction	6 TCMs	Stable phase	- N = 66 - Control group (conventional WM, expectorant, anti-infection, postural drainage, azithromycin, fodosteine) versus experimental group (control therapy plus *Juqin* decoction) - 2-month treatment	- Higher total effective rate^*b^ - More significant reduction in clinical syndrome scores^*b^ - Shorter clinical symptom resolution time^*b^ - Greater improvement in pulmonary function parameters (FEV_1_, FEV_1_%, FVC, FVC%, FEV_1_/FVC, MVV%)^*b^	[132]
*Sangbaipi* decoction	8 TCMs	Bronchiectasis with co-existing infection	- N = 62 - Control group (basic WM therapy) versus experimental group (basic WM therapy plus *Sangbaipi* decoction granules) - 1-month treatment	- Greater improvement in SGRQ scores at 3-month post-treatment^*b^	[133]
*Kuandonghua* san	10 TCMs	Acute exacerbation	- N = 100 - Control group (conventional WM) versus experimental group (conventional WM plus *Kuandonghua* san) - 7-day treatment	- Marked reduction in TCM symptom scores (cough, sputum volume/color/consistency, thirst, dry stool, yellow urine)^*a^ - Higher total effective rate (92% vs. 80%)^*a^ - More significant improvement in hematological parameters (WBC, GRAN, NLR)^*a^ - Greater reduction in systemic inflammation markers (ESR, CRP, PCT)^*a^ - Better arterial blood gas outcomes (increased PaO₂, decreased PaCO₂)^*a^	[134]
			- N = 116 - Control group (conventional WM) versus experimental group (conventional WM plus modified *Kuandonghua* sanr) - 7-day treatment	- Higher total effective rate^*b^ - Better arterial blood gas parameters (increased PaO₂, decreased PaCO₂)^*b^ - More significant reduction in leukocyte parameters (WBC, NEUT, NLR)^*b^ - Greater decrease in systemic inflammation markers (ESR, CRP, PCT)^*b^	[135]
*Xuanfei Huatan Sanyu* decoction	13 TCMs	Acute exacerbation	- N = 82 - Control group (conventional WM) versus experimental group (conventional WM plus *Xuanfei Huatan Suayu* decoction) - 2-week treatment	- Greater improvement in pulmonary function (FEV_1_, FVC, DLCO)^*b^ - More significant reduction in inflammatory markers (WBC, IL-6, LT-B4, hs-CRP)^*b^ - Better quality of life outcomes (SGRQ domain scores)^*b^ - Higher total effective rate*b	[136]
Modified *Huanglian Wendan* decoction	9 TCMs	Acute exacerbation	- N = 61 - Control group (anti-infection, expectorant) versus experimental group (control therapy plus modified *Huanglian Wendan* decoction) - 10-day treatment	- Higher total effective rate (93.3% versus 80.6%)^*b^ - More significant reduction in leukocyte parameters (WBC, NEUT, LYM)^*b^ - Increased hemoglobin levels^*b^	[137]
			- N = 78 - Control group (standard internal medicine) versus experimental group (control therapy plus modified *Huanglian Wendan* decoction) - 10-day treatment	- Higher total effective rate^*b^ - Greater improvement in pulmonary function (FVC, FEV_1_)^*b^ - More significant reduction in symptom scores (cough, sputum, hemoptysis)^*b^	[138]
			- N = 82 - Control group (anti-infection, expectorant) versus experimental group (control therapy plus modified *Huanglian Wendan* decoction) - 10-day treatment	- Higher total effective rate (92.68% versus 78.05%)^*b^ - Reduced leukocyte parameters (WBC, NEUT, LYM)^*b^	[139]
*Qingjin Beilou* decoction	12 TCMs	Acute phase	- N = 78 - Control group (conventional WM) versus experimental group (conventional WM plus *Qingjin Beilou* decoction) - 12-day treatment	- Superior clinical response rate (97.5% versus 78.95%)^*b^ - Marked alleviation of key symptoms (cough, expectoration, fever) and physical signs (moist rales) ^*a^ - Enhanced normalization of inflammatory and hematological markers (NEUT, CRP) with improved oxygenation (SaO₂, PaO₂) ^*a^	[140]
	14 TCMs		- N = 99 - Control group (conventional WM) versus experimental group (conventional WM plus *Qingjin Beilou* decoction) - 1-month treatment	- Higher total effective rate (98.00% versus 85.71%)^*b^ - More pronounced reduction in pro-inflammatory cytokines (IL-6, IL-1β, TNF-α)^*b^ - Greater improvement in quality of life scores (activity limitation, respiratory symptoms, disease impact)^*b^ - More significant alleviation of TCM symptoms (cough, dyspnea, hemoptysis, chest tightness)^*b^ - Superior enhancement in pulmonary function (FEV_1_, FVC, DLCO%pred)^*b^	[141]
			- N = 80 - Control group (conventional WM) versus experimental group (conventional WM) plus *Qingjin Beilou* decoction) - 12-day treatment	- Higher total effective rate (92.5% versus 67.5%)^*b^ - Superior improvement in pulmonary function (FEV_1_, FVC)^*b^ - More significant reduction in TCM syndrome scores and serum inflammatory cytokines (IL-6, IL-1β, TNF-α)^*b^	[142]
*Huangqin Xiebai* decoction	11 TCMs	Acute exacerbation	- N = 80 - Control group (conventional WM) versus experimental group (conventional WM plus *Huangqin Xiebai* decoction) - 10-day treatment	- Greater improvement in pulmonary function (FEV_1_, FEV_1_/FVC)^*b^ - More pronounced reduction in inflammatory markers (CRP, IL-6, TNF-α)^*b^	[143]
*Jianpi Yifei* decoction	16 TCMs	Stable phase	- N = 73 - Control group (conventional anti-infection, anti-inflammatory, bronchodilator) versus experimental group (control therapy plus *Jianpi Yifei* decoction) - 4-week treatment	- Higher total effective rate (89.47% versus 68.57%)^*b^ - Greater reduction in TCM syndrome scores^*b^ - Enhanced improvement in pulmonary function (FEV_1_, FVC, DLCO)^*b^ - More significant decrease in serum inflammatory markers (WBC, GRAN, CRP)^*b^	[144]
*Shenzhu Yangfei* decoction	15 TCMs	Stable phase	- N = 115 - Control group (WM symptomatic therapy) versus experimental group (control therapy plus *Shenshu Yangfei* decoction, *Kechuan* plaster) - 1-month treatment	- Higher total effective rate (92.86% versus 78.57%)^*b^ - Greater reduction in dyspnea scores (MMRC)^*b^ - Shorter symptom resolution time (cough, moist rales, expectoration)^*b^ - More pronounced decrease in inflammatory mediators (MMP-9, IL-6, IL-8)^*b^ - Enhanced lung function parameters (FEV1/FVC, IC, FEV_1_%pred)^*b^	[145]
*Cangma* pill	4 TCMs	Stable phase	- N = 66 - Control group (roxithromycin and ambroxol tablet) versus experimental group (control therapy plus low-dose *Cangma* pill) - 3-month treatment	- Higher total effective rate^*b^ - Reduced dyspnea scores^*b^ - Decreased 24-h sputum volume^*b^ - Improved quality of life scores^*b^	[146]
Modified *Cangma* pill	10 TCMs	Acute exacerbation	- N = 68 - Control group (oxygen, anti-infection, electrolyte correction, antitussive/expectorant, salbutamol aerosol, oral ambroxol) versus experimental group (control therapy plus modified *Cangma* pill) - 2-week treatment	- More significant reduction in TCM phlegm-dampness syndrome scores (white sputum, thick sputum quality, epigastric fullness, poor appetite, general heaviness) ^*a^ - Greater improvement in pulmonary function parameters (FEV_1_, FVC, FEV_1_/FVC) ^*a^ - Higher total effective rate (94.12% versus 70.59%)^*b^ - More pronounced decrease in serum inflammatory markers (CRP, WBC, NEUT%) ^*a^	[73]
Gleditsiae pill	2 TCMs	Acute exacerbation	- N = 62 - Control group (WM anti-infection) versus experimental group (control therapy plus gleditsiae pill) - 14-day treatment	- Higher total effective rate (96.87% versus 73.33%)^*b^ - Greater improvement in pulmonary function parameters (FEV_1_, FEV_1_%)^*b^ - More significant reduction in clinical symptom scores (cough, etc.)^*b^	[75]
*Bufei Ejiao* decoction	9 TCMs	Acute exacerbation	- N = 86 - Control group (phentolamine, anti-infection, antitutsive, expectorant, sedative) versus experimental group (control therapy plus *Bufei Ejiao* decoction) - 1-week treatment	- Longer 6-min walk distance and higher sputum clearance volume^*b^ - Lower dyspnea scores and enhanced quality of life across multiple domains (physical, psychological, independence, environmental, social)^*b^ - Reduced incidence of adverse reactions^*b^	[147]
*Baihe Gujin* decoction	9 TCMs	Bronchiectasis with co-existing infection	- N = 92 - Control group (ambroxol OD, theophylline SR, ofloxacin) versus experimental group (control therapy plus *Baihe Gujin* decoction) - 1-month treatment	- Higher total effective rate (95.65% versus 82.61%)^*b^ - Shorter symptom resolution time (dry throat, cough, hemoptysis, tidal fever)^*b^ - Greater improvement in oxygenation (SaO₂) and lung function (FVC)^*b^	[148]
			- N = 80 - Control group (ambroxol, ofloxacin, theophylline SR) versus experimental group (control therapy plus modified *Baihe Gujin* decoction) - 4-week treatment	- More favorable total effective rate for Yin deficiency lung heat pattern bronchiectasis with infection^*b^ - Enhanced clinical parameters (SaO₂, CRP, FVC) post-intervention^*b^ - Shorter duration of key symptoms (dry throat, cough, tidal fever, hemoptysis)^*b^	[85]
Modified *Wanfei* yin	13 TCMs	Bronchiectasis with co-existing infection	- N = 70 - Control group (conventional symptomatic therapy) versus experimental group (experimental group plus modified *Wanfei* yin) - 2-week treatment	- Higher total effective rate (94.29% versus 71.43%)^*b^ - More substantial reduction in TCM symptom scores (dry cough, fatigue, spontaneous sweating, hot palms/soles)^*b^Enhanced pulmonary function parameters (PEF, FEV_1_, FEV_1_%pred)^*b^	[83]

^*a^Experimental group versus control group, P < 0.01

^*b^Experimental group versus control group, P < 0.05

*FEV1* Forced expiratory volume in 1 s, *FVC* forced vital capacity, *FEV1/FVC* the ratio of FEV1 to FVC, *FVC%pred* the percentage of forced vital capacity, *FEV1%pred* the percentage of FEV1, *PEF* the peak expiratory flow, *DLCO%pred* the percentage of diffuse capacity for carbon monoxide, *IC* the inspiratory capacity, *6MWT* 6-min walk test, *mMRC* modified Medical Research Council dyspnea scale, *BODE* body mass index, airflow obstruction, dyspnea, and exercise capacity index, *SGRQ* St. George’s Respiratory Questionnaire, *WBC* white blood cell count, *NEUT* neutrophil count, *LYM* lymphocyte count, *NLR* neutrophil-to-lymphocyte Ratio, *GRAN* granulocyte count, *CRP* C-reactive protein, *hs-CRP* high-sensitivity C-reactive protein, *PCT* procalcitonin, *ESR* erythrocyte sedimentation rate, *CD3⁺* cluster of differentiation 3-positive T cells, *CD4⁺* cluster of differentiation 4-positive T helper cells, *CD8⁺* cluster of Differentiation 8-positive T cytotoxic cells, *CD4*^*+*^*/CD8*^*+*^ the ratio of CD4-positive to CD8-positive T cells, *TNF-α* tumor necrosis factor-alpha, *IL-2* interleukin-2, *IL-4* interleukin-4, *IL-6* interleukin-6, *IL-8* interleukin-8, *IL-1β* interleukin-1 beta, *TGF-β* transforming growth factor-beta, *LT-B4* leukotriene B4, *MUC5AC* mucin 5AC, *NE* neutrophil elastase, *MMP-9* matrix metalloproteinase-9, *TIMP-1* tissue inhibitor of metalloproteinase-1, *BALF* bronchoalveolar lavage fluid, *PaO*₂ the arterial partial pressure of oxygen, *PaCO*₂ the arterial partial pressure of carbon dioxide, *SaO*₂ the arterial oxygen saturation.

The integration of specific TCM formulae with standard WM regimens has been a focus of research. For example, combining *Qingjin Huatan* decoction with antibiotics has been associated with benefits extending beyond antimicrobial action. These results revealed enhanced infection clearance alongside significant improvements in pulmonary function parameters (FEV_1_, FVC, and PEF), a reduction in systemic inflammatory markers (WBC, CRP, and PCT), and modulation of the local inflammatory environment through decreased sputum cytokine levels (TNF-α, IL-6, and IL-8). Furthermore, this integrated approach demonstrated immunomodulatory effects characterized by elevated IL-2 with concurrent reduction in the levels of TGF-β, IFN-γ, and interleukins (IL-23, IL-4, and IL-5), and improved immune function, as evidenced by increased CD4^+^ and CD4^+^/CD8^+^ ratios [120, 122]. This multitargeted therapeutic strategy suggests that integrated therapy may target not only infection but also the underlying inflammatory and immune dysregulation in bronchiectasis.

**Fig. 5:**
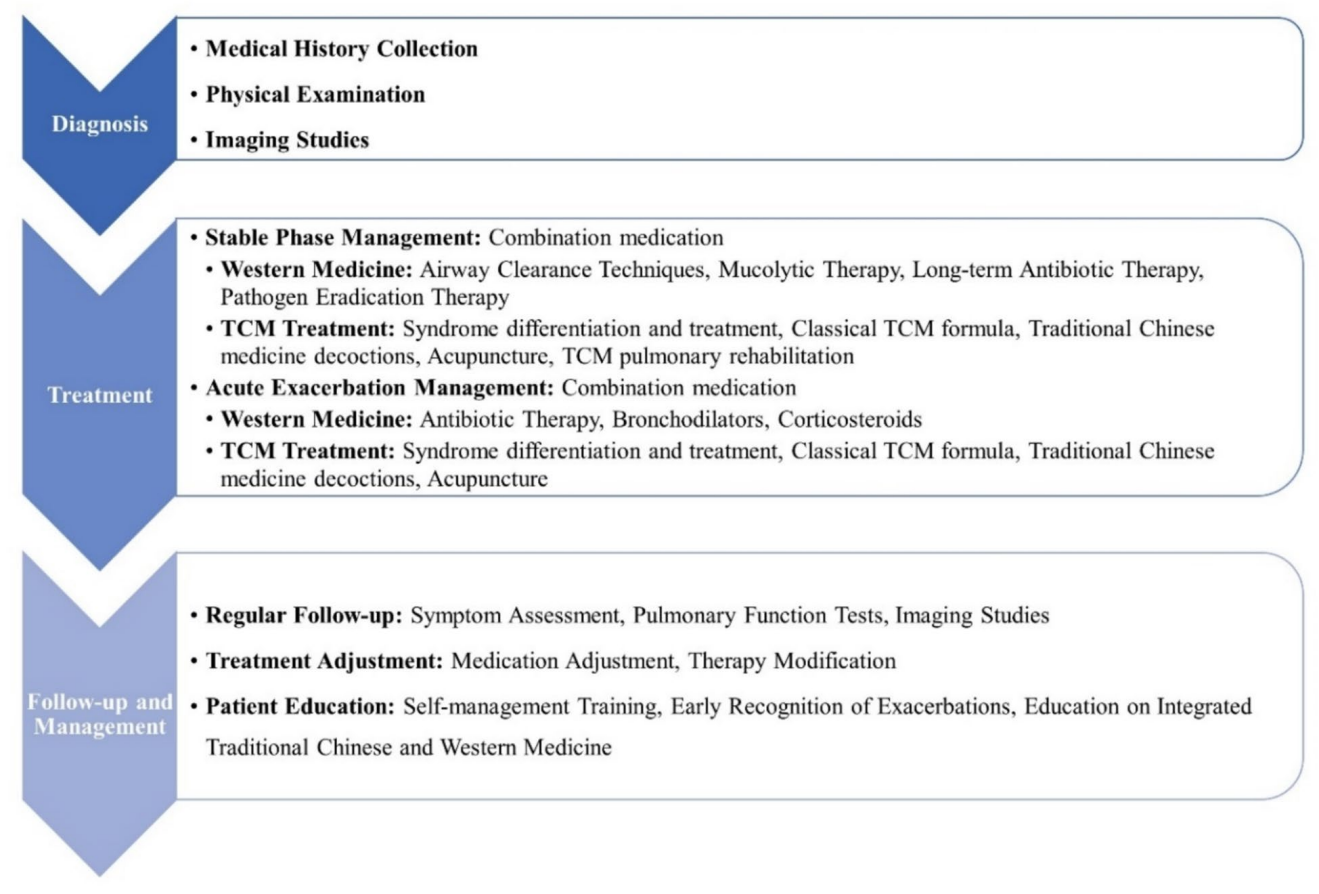
Flowchart of disease management for bronchiectasis. The disease management flowchart for bronchiectasis provides a comprehensive framework for diagnosing, treating, and following patients with the disease. These findings emphasize the importance of individualized treatment plans and patient education in managing this chronic respiratory disease, incorporating both TCM and WM approaches

Current expert consensus recommends phenotype-targeted personalized strategies and advises the selection of TCM formulae and WM agents according to patient-specific characteristics, including sputum patterns, infection types, and physiological profiles [150]. Figure 5 provides a systematic implementation framework for these integrated principles, offering clinicians a structured approach to patient management. This personalized methodology enables more effective addressing of bronchiectasis heterogeneity. For example, patients with dominant neutrophilic inflammation might be considered for a regimen combining macrolide antibiotics with TCM herbs possessing putative immunomodulatory properties [151], whereas those with impaired mucus clearance may achieve superior outcomes through synergistic application of mucolytics and phlegm-resolving drugs such as *P. ternata*. By systematically integrating diagnostic findings with both TCM and WM assessment parameters, clinicians can develop tailored treatment plans that address both the pathological processes and the individual’s constitutional characteristics, aiming to leverage the complementary strengths of each system.

### Future perspective

The advancement of personalized treatment strategies reflects a deeper recognition of individual variability among patients with bronchiectasis. Such strategies consider factors such as the patient’s genetic background, environmental exposure, lifestyle, and complications, aiming to provide the most suitable treatment plan for each patient. While symptoms may appear similar, individuals often demonstrate vast differences in disease progression, complications, and inflammatory markers. Therefore, some researchers believe that each patient can be viewed as a Rubik’s cube and that solving any Rubik’s cube requires "observation" from all directions because it is necessary to understand the content of all other faces. Modern multiomics technologies, spanning genomics, epigenomics, transcriptomics, metabolomics, lipidomics, and microbiomics, provide precisely these critical perspectives, enabling the formulation of truly individualized management plans on the basis of a complete picture of the patient [152].

Bronchiectasis continues to present substantial therapeutic challenges owing to its etiological diversity and pathophysiological complexity. The limitations of conventional approaches are particularly evident in antibiotic management. Individual variations in infectious triggers, inflammation, and structural remodeling processes create intricate management requirements that demand specific solutions. Furthermore, environmental determinants such as air pollutants are recognized as influential factors, although their direct causal links to outcomes require further clarification.

TCM has demonstrates distinctive in terms of acute symptom control and stable phase prolongation, but further mechanistic elucidation and standardization of efficacy assessment are needed. Future investigations should prioritize the identification of novel therapeutic targets, the optimization of integrated protocols, and enhanced clinical research methodologies for TCM-WM treatment. These efforts should ultimately aim to improve both disease remission rates and the quality of life of patients.

Integrated TCM-WM treatment represents a multidimensional therapeutic strategy addressing key clinical challenges in bronchiectasis management, including reduced antibiotic dependence and resistance, decreased acute exacerbation frequency, attenuated pulmonary function decline, and enhanced host defense mechanisms during stable disease phases. Building upon these preliminary findings, future research should focus on two pivotal directions to advance this field. First, incorporating systems biology approaches is needed to elucidate the potential synergistic mechanisms between TCM and WM, which would provide a more robust scientific foundation for personalized treatment in the precision medicine era. Second, and fundamentally, conducting high-quality, multicenter RCTs is essential to definitively establish the efficacy, safety, and optimal protocols for integrated TCM-WM treatment, thereby strengthening the overall robustness of the clinical evidence base [153].

In summary, bronchiectasis is a prototypical heterogeneous disease strongly influenced by individual factors and multiple interacting pathways. Owing to individual differences in pathogenic factors and other aspects among patients, the clinical efficacy of single therapy is not ideal. The integration of TCM and WM represents a promising, multidimensional strategy for the management of bronchiectasis, which may offer a more comprehensive approach to improve clinical efficacy and patients’ quality of life. This review outlines current options, highlights the advantages of combined treatment, and proposes disease management recommendations for combined treatment, laying the foundation for future research and clinical practice. Further research is needed in the future to optimize the treatment plan and verify the long-term benefits of combining TCM and WM.

## Data Availability

No datasets were generated or analysed during the current study.

## Abbreviations

ABPA: Allergic bronchopulmonary aspergillosis
APS: Astragalus polysaccharides
CCL1: C-C Motif chemokine ligand 1
CD4^+^: Cluster of differentiation 4-positive T cells
CD8^+^: Cluster of differentiation 8-positive T cells
CD86: Cluster of differentiation 86
CF: Cystic fibrosis
CFTR: Cystic fibrosis transmembrane conductance regulator
COPD: Chronic obstructive pulmonary disease
COX-2: Cyclooxygenase-2
CPMs: Chinese patent medicines
CRP: C-Reactive protein
CXCL1: Chemokine (C-X-C Motif) ligand 1
CVID: Common variable immunodeficiency
E-cadherin: Epithelial cadherin
EMT: Epithelial-mesenchymal transition
EPO: Erythropoietin
ERK: Extracellular signal-regulated kinase
ET: Exercise training
FEV1: Forced expiratory volume in one second
FVC: Forced vital capacity
GERD: Gastroesophageal reflux disease
ICS: Inhaled corticosteroid
IFN-γ: Interferon gamma
IL-1β: Interleukin 1 beta
IL-4: Interleukin 4
IL-5: Interleukin 5
IL-6: Interleukin 6
IL-8: Interleukin 8
IL-13: Interleukin 13
IL-17: Interleukin 17
IL-23: Interleukin 23
LPS: Lipopolysaccharide
MBP: Major basic protein
MRC1: Mannose receptor C-type 1
NAC: N-Acetylcysteine
NSAIDs: Nonsteroidal anti-inflammatory drugs
PCT: Procalcitonin
PEF: Peak expiratory flow
PNEC: Pulmonary neuroendocrine cell
PR: Pulmonary rehabilitation
RCTs: Randomized controlled trials
SnoN: SnoN transcriptional co-repressor
TCM: Traditional Chinese medicine
TGF-β: Transforming growth factor beta
TNF-α: Tumor necrosis factor alpha
WBC: White blood cell count
WM: Western medicine
